# Signal transducer and activator of transcription 2 deficiency is a novel disorder of mitochondrial fission

**DOI:** 10.1093/brain/awv182

**Published:** 2015-06-30

**Authors:** Rojeen Shahni, Catherine M. Cale, Glenn Anderson, Laura D. Osellame, Sophie Hambleton, Thomas S. Jacques, Yehani Wedatilake, Jan-Willem Taanman, Emma Chan, Waseem Qasim, Vincent Plagnol, Annapurna Chalasani, Michael R. Duchen, Kimberly C. Gilmour, Shamima Rahman

**Affiliations:** ^1^ 1 Mitochondrial Research Group, Genetics and Genomic Medicine, UCL Institute of Child Health, Guilford Street, London, UK; ^2^ 2 Molecular Immunology Unit, Great Ormond Street Hospital, London, UK; ^3^ 3 Histopathology Unit, Great Ormond Street Hospital, London, UK; ^4^ 4 Department of Biochemistry and Molecular Biology, Monash University, Melbourne 3800, Australia; ^5^ 5 Primary Immunodeficiency Group, Institute of Cellular Medicine, Newcastle University, UK; ^6^ 6 Developmental Neurosciences, UCL Institute of Child Health, London, UK; ^7^ 7 Department of Clinical Neurosciences, UCL Institute of Neurology, Rowland Hill Street, London, UK; ^8^ 8 UCL Genetics Institute, London, UK; ^9^ 9 Neurometabolic Unit, National Hospital for Neurology and Neurosurgery, London, UK; ^10^ 10 Cell and Developmental Biology, University College London, UK; 11 11 Metabolic Unit, Great Ormond Street Hospital, London, UK

**Keywords:** STAT2, dynamin-related protein 1 (DRP1), mitochondrial disease, JAK-STAT signalling, mitochondrial fission, mitochondrial fusion

## Abstract

**See Dasgupta et al. (doi:10.1093/awv237) for a scientific commentary on this article.**

Defects of mitochondrial dynamics are emerging causes of neurological disease. In two children presenting with severe neurological deterioration following viral infection we identified a novel homozygous *STAT2* mutation, c.1836 C>A (p.Cys612Ter), using whole exome sequencing. In muscle and fibroblasts from these patients, and a third unrelated STAT2-deficient patient, we observed extremely elongated mitochondria. Western blot analysis revealed absence of the STAT2 protein and that the mitochondrial fission protein DRP1 (encoded by *DNM1L*) is inactive, as shown by its phosphorylation state. All three patients harboured decreased levels of DRP1 phosphorylated at serine residue 616 (P-DRP1^S616^), a post-translational modification known to activate DRP1, and increased levels of DRP1 phosphorylated at serine 637 (P-DRP1^S637^), associated with the inactive state of the DRP1 GTPase. Knockdown of *STAT2* in SHSY5Y cells recapitulated the fission defect, with elongated mitochondria and decreased P-DRP1^S616^ levels. Furthermore the mitochondrial fission defect in patient fibroblasts was rescued following lentiviral transduction with wild-type *STAT2* in all three patients, with normalization of mitochondrial length and increased P-DRP1^S616^ levels. Taken together, these findings implicate STAT2 as a novel regulator of DRP1 phosphorylation at serine 616, and thus of mitochondrial fission, and suggest that there are interactions between immunity and mitochondria. This is the first study to link the innate immune system to mitochondrial dynamics and morphology. We hypothesize that variability in JAK-STAT signalling may contribute to the phenotypic heterogeneity of mitochondrial disease, and may explain why some patients with underlying mitochondrial disease decompensate after seemingly trivial viral infections. Modulating JAK-STAT activity may represent a novel therapeutic avenue for mitochondrial diseases, which remain largely untreatable. This may also be relevant for more common neurodegenerative diseases, including Alzheimer’s, Huntington’s and Parkinson’s diseases, in which abnormalities of mitochondrial morphology have been implicated in disease pathogenesis.

## Teaser

Shahni *et al.* identify a novel mutation in *STAT2*, which encodes a component of the JAK-STAT cytokine signalling pathway, in three patients with severe neurological deterioration following viral infection. STAT2 is shown to regulate mitochondrial fission, suggesting a new avenue for treatment of mitochondrial diseases and possibly common neurodegenerative disorders.

## Graphical abstract

**Figure:**
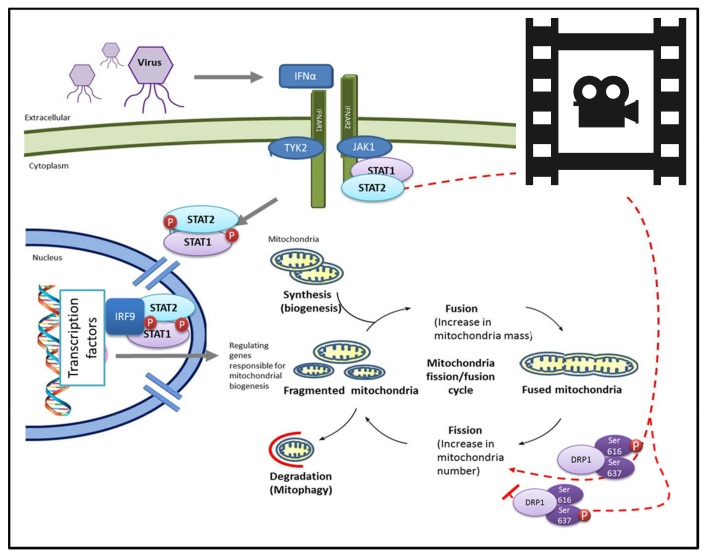
**See Dasgupta et al. (doi:10.1093/awv237) for a scientific commentary on this article.** Shahni *et al.* identify a novel mutation in *STAT2*, which encodes a component of the JAK-STAT cytokine signalling pathway, in three patients with severe neurological deterioration following viral infection. STAT2 is shown to regulate mitochondrial fission, suggesting a new avenue for treatment of mitochondrial diseases and possibly common neurodegenerative disorders.

## Introduction

Mitochondrial diseases are clinically, biochemically and genetically heterogeneous disorders characterized by dysfunction of mitochondria, which are highly dynamic organelles with multitudinous functions including energy generation by oxidative phosphorylation (OXPHOS). Although the dynamic nature of mitochondria was recognized nearly 100 years ago, only recently have the processes determining mitochondrial morphology been unravelled (Lewis and Lewis, 1914; Archer, 2013). Mitochondrial networks are defined by a delicate balance of fusion and fission events that link mitochondrial biogenesis to mitophagy, a quality control process for removing dysfunctional mitochondria in the mitochondrial ‘life cycle’. When this balance is perturbed, mitochondrial disease ensues (Table 1) (Chan, 2006; Youle and van der Bliek, 2012).

**Table 1:** Molecular defects leading to disturbed mitochondrial dynamics

Genetic defect	Mechanism	Clinical features
*DNM1L* (DRP1)	Impaired fission	Fatal infantile encephalopathy (cardiomyopathy in a mouse model)
*MFF* (mitochondrial fission factor)	Impaired fission	Encephalopathy and neuropathy
*MFN2* (mitofusin 2)	Impaired fusion	Charcot–Marie–Tooth disease type 2 A (peripheral neuropathy, sometimes with optic atrophy)
*OPA1*	Impaired fusion	Autosomal dominant optic atrophy
*PINK1* (PTEN-induced kinase 1)	Impaired mitophagy	Autosomal recessive juvenile parkinsonism
*PARK2* (parkin)	Impaired mitophagy	Autosomal recessive juvenile parkinsonism

Several factors necessary for mitochondrial fusion and fission have been identified (Archer, 2013). Mitochondrial fusion is mediated by several large membrane guanosine triphosphatases (GTPases): the mitofusins (MFN1 and MFN2) on the outer membrane, and optic atrophy 1 (OPA1) on the inner membrane. Factors involved in fission include dynamin related protein 1 (DRP1, encoded by *DNM1L*), receptor proteins MiD49 (*MIEF2*) and MiD51 (*MIEF1*), mitochondrial fission protein 1 (*FIS1*) and mitochondrial fission factor (*MFF*) (Palmer *et al.*, 2011; Losón *et al.*, 2013). However, the exact processes coordinating fission and fusion remain unknown.

Previously mutations in the fission factors DRP1 and MFF have been implicated in causing human mitochondrial fission defects (Waterham *et al.*, 2007; Shamseldin *et al.*, 2012). We have now used whole exome sequencing to identify mutations in *STAT2*, encoding a component of the JAK (janus kinase)-STAT (signal transduction and activation of transcription) cytokine signalling pathway (Steen and Gamero, 2013), as a novel cause of disrupted mitochondrial fission in three patients from two unrelated pedigrees. These data are the first to implicate signalling pathways of the innate immune system in the regulation of mitochondrial dynamics.

## Materials and methods

### Patients

Patient 1 is the elder child of non-consanguineous Albanian parents. He was well and developing normally until 12 months of age when he developed a febrile illness 1 week after mumps, measles and rubella (MMR) vaccination unresponsive to antibiotics. CSF cell count was normal. Pyrexia, lethargy, conjunctivitis, inflamed throat and lymphadenopathy continued, leading to a diagnosis of atypical Kawasaki disease, treated with intravenous immunoglobulin. Measles serology prior to immunoglobulin administration was consistent with recent MMR vaccination. C-reactive protein (CRP) was initially raised but rapidly fell to normal levels.

One month later, he was readmitted with opsoclonus-myoclonus. EEG and brain MRI were normal, but repeat CSF examination revealed 12 × 10^6^/l lymphocytes and low glucose, with negative bacterial cultures and viral PCRs. He was treated empirically with intravenous acyclovir and steroids, and improved. Immunology investigations demonstrated low CD4-positive T cells. Major Histocompatibility Complex II deficiency and HIV were excluded, and proliferative response to phytohaemagglutinin was normal. He remained well until 2.5 years when he presented with fever, diarrhoea, hypoglycaemia, impaired renal and liver function, metabolic acidosis (pH 7.15), hyperammonaemia, markedly elevated CRP and thrombocytopaenia (Supplementary Table 1). He then developed opsoclonus-myoclonus and seizures, with neuroimaging suggestive of meningoencephalitis. He received treatment with antimicrobials, antifungals and steroids. Seizures were very difficult to control, and he developed significant neurological impairment including four-limb spasticity, chorea and severe cortical visual impairment, which has not recovered. He remains on triple anticonvulsant therapy, replacement immunoglobulin and antibiotic prophylaxis.

Patient 2, the younger sister of Patient 1, was well until aged 13 months when she developed a febrile illness 1 week after MMR vaccine. She was anaemic and lymphopaenic with deranged clotting. CSF was normal except for a positive PCR for mumps virus. Throat swab PCR was positive for measles, mumps and rubella. She had detectable antibodies to all three viruses, suggesting an appropriate immunological response to the vaccine (Supplementary Table 1). She was treated with intravenous acyclovir, and was readmitted aged 15 months with persistent fever and malaise. She developed septic shock with metabolic acidosis requiring intensive care. CSF culture and PCR for herpes simplex virus (HSV), varicella zoster virus (VZV) and enterovirus were negative. She was treated with intravenous ceftriaxone, clindamycin and acyclovir. Her blood counts normalized, and bone marrow aspirate and lymph node biopsy showed no haemophagocytic lymphohistiocytosis. Autoantibodies were negative. She made a slow recovery with no neurological or other deficit, and has remained well other than a diarrhoeal illness, during which immunological testing revealed marked lymphopaenia with low B and T cells. Over the next 12 months her lymphocyte count and T and B cell numbers normalized. Due to the apparent association with infection, she remains on replacement immunoglobulin. In addition to neurological and immune dysfunction, both children had evidence of mild renal tubulopathy. Detailed clinical data are provided in Supplementary Table 1.

Patient 3 (Hambleton *et al.*, 2013), from an unrelated family, was reported previously to have experienced a prolonged febrile illness with multisystem involvement following MMR. Her affected cousin developed a sterile encephalitic illness following the same vaccine (Hambleton *et al.*, 2013).

### Histological analysis of mitochondrial morphology in skeletal muscle and fibroblasts

#### Electron microscopy

Open skeletal muscle and skin biopsies were performed after informed parental consent. For ultrastructural examination of skeletal muscle and cultured skin fibroblasts, samples were fixed in 2.5% glutaraldehyde buffered with 0.1 M sodium cacodylate (pH 7.2), postfixed in 1% osmium tetroxide, dehydrated in ascending grades of alcohol, processed through propylene oxide and embedded in Epon^™^ resin. Ultrathin sections were cut with a diamond knife on a Leica Ultracut UCT Ultramicrotome, placed on copper grids and stained with uranyl acetate and lead citrate. Examination was carried out with a JEOL 1400 transmission electron microscope.

#### Live cell imaging

Live cell imaging using confocal microscopy was used to visualize tetramethyl rhodamine methyl ester (TMRM)-labelled mitochondria in fibroblasts. Primary skin fibroblasts cultured under standard conditions as described previously (Fassone *et al.*, 2010) were labelled with 25 nM TMRM (Invitrogen) prior to visualizing mitochondria with the use of a Zeiss LSM 700 ×63 oil immersion objective. Identical detector/gain settings were used for all samples. To determine membrane potential, mean fluorescence intensity (background corrected) of TMRM-labelled mitochondria was analysed using ImageJ (*n =* 3; >27 cells analysed for each *n*).

### Genetic analyses

#### Mitochondrial DNA analysis

The whole mitochondrial genome was sequenced in muscle from Patient 1 using Sanger sequencing, and mtDNA copy number per diploid nuclear genome was determined in patient and control fibroblasts by Droplet Digital^™^ PCR (Bio-Rad). The primers and probes used in this study have been published previously (Bai and Wong, 2005).

#### Candidate nuclear gene sequencing

Candidate nuclear genes (*DRP1*, *MFF* and *FIS1*) previously implicated in mitochondrial fission were analysed by Sanger sequencing of DNA extracted from blood from Patients 1 and 2.

#### Homozygosity mapping

Genome-wide SNP (single nucleotide polymorphism) array was performed using Illumina HumanCytoSNP 12 in Patient 1, Patient 2 and both parents to search for regions of homozygosity by descent that might contain the mutated gene.

#### Whole exome sequencing and filtering criteria

The whole exome was sequenced in Patients 1 and 2 using the Illumina HiSeq 2000 platform. Sample preparation and enrichment was performed according to Agilent's SureSelect Protocol Version 1.2. Concentration of each library was determined using Agilent's QPCR NGS Library Quantification Kit (G4880A). Samples were pooled prior to sequencing, with each sample at a final concentration of 10 nM. Read files (Fastq) were generated from the sequencing platform via runs to an average 50× coverage on an in-house HiSeq 2000 system (Illumina). Raw fastq files were aligned to the GRCh37 reference genome using novoalign version 2.08.03. Duplicate reads were marked using Picard tools MarkDuplicates. Calling was performed using the haplotype caller module of GATK (https://www.broadinstitute.org/gatk, version 3.1-1), creating gVCF formatted files for each sample. The individual gVCF files for the exomes discussed in this study, in combination with ∼3000 clinical exomes (UCL-exomes consortium), were combined into merged VCF files for each chromosome containing on average 100 samples each. The final variant calling was performed using the GATK GenotypeGVCFs module jointly for all samples (cases and controls). Variant quality scores were then recalibrated according to GATK best practices separately for indels and SNPs. Resulting variants were annotated using ANNOVAR. Candidate variants were filtered based on function (non-synonymous, presumed loss-of-function or splicing) and minor allele frequency (<0.5% minor allele frequency in our internal control group, as well as the NHLBI exome sequencing data set).

### *STAT2* lentiviral transduction and silencing

#### *STAT2* transduction

Lentiviral transduction of patient fibroblasts with wild-type *STAT2* was performed to determine whether the mitochondrial fission defect could be rescued by exogenous wild-type *STAT2.* The human *STAT2* gene was cloned into the self-inactivating HIV1-derived lentiviral vector plasmid as previously described (Demaison *et al.*, 2002). The plasmid uses the spleen focus-forming virus promoter to drive expression of the *STAT2.* Expression was linked to enhanced green fluorescent protein through an internal ribosomal entry site.

HEK293T cells were cultured in Dulbecco’s modified Eagle medium (Invitrogen) supplemented with 10% foetal calf serum (Sigma). These cells were seeded in T175 flasks and grown overnight to reach 80% confluency. Each flask was transfected with 50 µg vector plasmid, 17.5 µg vesicular stomatitis virus (VSV) envelope plasmid (pMDG), and 32.5 µg *gag/pol* packaging plasmid (pCMVΔ8.74) using 2.5 nM polyethylenimine. Viral supernatant was harvested 48 and 72 h after transfection, filtered at 0.22 μm and concentrated by ultracentrifugation at 23 000*g* for 2 h at 4°C using a Beckmann ultracentrifuge. Virus particles were resuspended in Opti-Mem^®^ and stored at −80°C. The number of viral infectious particles was calculated by flow cytometry to measure GFP in HEK293T cells, 72 h after transduction with serial dilutions of virus.

#### *STAT2* silencing

Silencing the *STAT2* gene in SHSY5Y cells was achieved by transducing the cells with the pGIPZ shRNAmir vector, as previously described (Fassone *et al.*, 2010). Stably transduced clones were selected in 1 µg/ml puromycin (Gibco-Invitrogen). *STAT2* knockdown efficiency was tested by quantitative PCR and immunoblot analyses.

### Cellular analyses

#### Transcript expression

Total RNA was extracted from patient and control cells using RNAqueous^®^-4PCR (ABI). For each sample, 1 μg of total RNA was reverse transcribed to cDNA using the high capacity RNA to cDNA kit (ABI). Oligonucleotide primers for quantitative real time PCR were designed using the universal probe library (Roche Molecular Biochemicals), and synthesized at Sigma-Genosys. Quantitative real time PCR was performed using the Power SYBR^®^ green mix (ABI) on a StepOne^™^ quantitative PCR machine (ABI). The target genes analysed were *DRP1*, *MFN1*, *MFN2* and *OPA1*, with *ACTB* (encoding β-actin) as a reference gene. Relative quantification of gene expression in patient samples was carried out against control 1 (C1).

#### Protein expression

For immunodetection of STAT2, DRP1 (and phospho-DRP1) and the fusion proteins MFN1, MFN2 and OPA1, cell pellets of fibroblasts cultured in Dulbecco’s modified Eagle medium containing GlutaMAX^™^-I and 50 mM glucose (Life Technologies) were extracted on ice in PBS, 1.5% n-dodecyl-β-d-maltoside. Equal concentrations of protein were denatured in Laemmli sample Buffer and 10 mM dithiotreitol, resolved on 4–15% Mini-Protean^®^ TGX Stain-Free^™^ gels with Precision Plus Protein standards and blotted onto 0.2 µm PVDF membranes with a Trans-blot^®^ Turbo^™^ Transfer System (all from Bio-Rad). Blots were blocked in PBS, 10% skimmed milk powder (Fluka) and incubated overnight with primary antibody. For the phospho-immunoblots, the primary antibodies used were: rabbit anti-Phospho-DRP1 (Ser616), rabbit anti-Phospho-DRP1 (Ser637), rabbit anti-DRP1 (D6C7), rabbit anti-STAT2 (Sigma) and anti-phosphorylated STAT1 antibodies (all from Cell Signaling). For western blots the primary antibodies used were: anti-DRP1 (BD Transduction Laboratories), anti-MFN1 (Abcam), anti-MFN2 (Abcam), anti-OPA1 (BD Transduction Laboratories), anti-MTCO2 (Abcam), anti-TOM20 (Santa Cruz Biotechnology) and anti-β-actin (Abcam). Blots were incubated with the appropriate secondary antibody (polyclonal goat anti-mouse or anti-rabbit IgG/HRP from Dako) and were developed for 5 min with Clarity^™^ Western ECL substrates (Bio-Rad) and visualized on a ChemiDoc^™^ MP imager (Bio-Rad).

#### DRP1 localization

Cells were grown on glass chamber slides (Ibidi), fixed with paraformaldehyde and incubated with anti-DRP1 primary antibody (Cell Signaling) overnight and anti-rabbit FITC conjugated secondary antibody (Sigma) for 1 h. Nuclei were stained with Hoechst and mitochondria were stained with anti-TOM20 (Santa Cruz Biotechnology). Cells were viewed under a Zeiss LSM 700 ×63 oil immersion objective using appropriate filters.

#### Fluorescence-activated cell sorting for detection of STAT1 phosphorylation

To detect STAT1 phosphorylation, native and transduced patient fibroblasts were either left unstimulated or stimulated with 10^5^ units/ml of IFNα (Stratech Scientific Limited). Cells were then lysed, fixed, washed and permeabilized before adding 5 μl anti-STAT1 phosphorylated tyrosine antibody (BD Bioscience) as previously described (Walshe *et al.*, 2009). Ten thousand fibroblasts were acquired and analysed (FACSCalibur and CELLQest software 2.1.1, BD Biosciences). The percentage change in phosphorylated cells was calculated by subtracting the percentage of unstimulated cells from that of the stimulated cells. Experiments were performed in triplicate and expression levels were expressed as mean ± standard error (SE).

#### Apoptosis assay

Apoptosis was analysed in fibroblasts derived from the STAT2 deficient index patient (Patient 1) and compared to fibroblasts derived from healthy individuals. Cultured fibroblasts were left unstimulated or stimulated with 100 U/ml IFNα (PBL Interferon Source), 10 µg/ml anti-fasL IgM (activating antibody from Millipore) to crosslink the fasL receptor or phytohaemagglutinin (10 µg/ml, BioStat). Five hours post stimulation, cells were stained with Annexin V (Life Technologies) and 7AAD (BD Biosciences). Ten thousand cells were acquired on a FACSCalibur and analysed using CellQuest Pro (BD Biosciences) and the percentage of apoptotic cells defined as annexin V+7AAD+.

## Results

### Clinical findings and laboratory analyses

The siblings Patients 1 and 2 presented with severe neurological deterioration following live attenuated viral immunization, and also had evidence of immune deficiency. The multisystem nature of their disease, together with mildly elevated plasma and CSF lactate levels in Patient 1 (Supplementary Table 1), led to clinical suspicion of a mitochondrial disorder. The elevated CSF neopterin levels observed in Patients 1 and 2 (Supplementary Table 1) were also compatible with an underlying mitochondrial disorder (Hasselmann *et al.*, 2010). Consequently open muscle biopsies were performed in both siblings. Spectrophotometric analysis of mitochondrial respiratory chain enzyme activities (OXPHOS complexes I–IV) was normal in skeletal muscle from Patients 1 and 2 (Supplementary Table 1) but in-gel activity of ATP synthase (complex V) was reduced on blue-native gel electrophoresis, particularly in Patient 1 who was clinically more severely affected (data not shown).

#### Histological analyses

**Figure 1:**
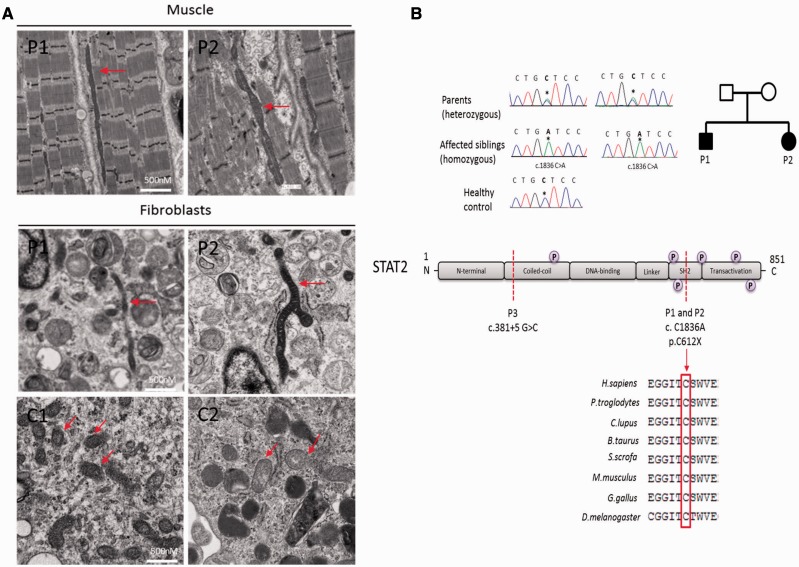
**Analysis of muscle and cultured skin fibroblasts.** (**A**) Representative electron micrographs of muscle (longitudinal sections, *top*) and fibroblasts (*bottom*) in patients and controls. Red arrows show mitochondria. (**B**) *Top*: Electropherograms showing Sanger sequence confirmation of novel homozygous stop gain mutation c.1836 C>A (p.Cys612Ter) in *STAT2* identified by whole exome sequencing in Patients 1 and 2. Parents are both heterozygous for the mutation. *Bottom*: The mutation leads to a stop-gain at amino acid position 612 of the highly conserved SH2 domain of STAT2 protein. P represents phosphorylation sites on *STAT2*.

Light microscopy of skeletal muscle was unremarkable but electron microscopy of muscle and fibroblasts from both children revealed abnormally long mitochondria, between 8–10 µm in length (Fig. 1A), suggesting defective mitochondrial fission.

### Genetic analyses

#### Homozygosity mapping and candidate gene analysis

Genome-wide SNP array revealed several 1 Mb regions of homozygosity shared by the siblings, and an extended region of homozygosity (10.6 Mb) on chromosome 12 (position: 52 648 837–63 282 047). Sanger sequencing excluded mtDNA mutations and mutations in candidate genes known or predicted to cause defective mitochondrial fission (*DRP1*, *MFF* and *FIS1*) (Waterham *et al.*, 2007; Shamseldin *et al.*, 2012; Shen *et al.*, 2014). Absolute quantification of mtDNA copy number per cell showed a 2-fold increase in Patient 1 compared to five paediatric controls [increased from a mean ± standard deviation (SD) of 658 ± 136 in controls to 1310 in Patient 1].

#### Whole exome analysis

Whole exome sequencing did not demonstrate any pathogenic mutations in known or putative mitochondrial disease genes in these siblings, but did reveal a novel homozygous stop-gain mutation c.1836 C > A (p.Cys612Ter) in *STAT2*, located within the region of extended homozygosity on chromosome 12.

#### Molecular and functional characterization of mutated STAT2

The homozygous c.1836 C > A (p.Cys612Ter) mutation, located within the SH2 domain of STAT2 which is critical for transcriptional function, was confirmed by Sanger sequencing and shown to be heterozygous in both parents (Fig. 1B).

### *STAT2* lentiviral transduction and silencing

#### *STAT2* transduction

**Figure 2:**
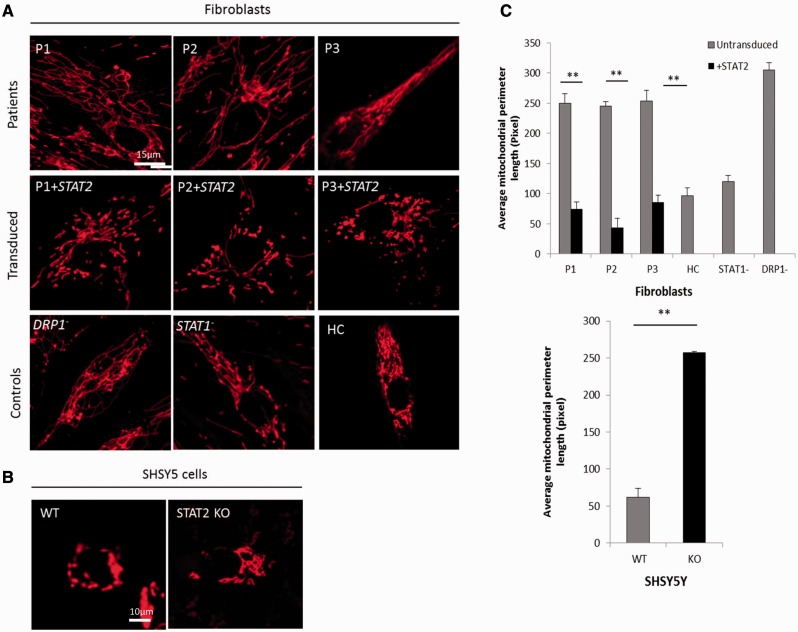
**Mitochondrial length analysis using TMRM staining and confocal microscopy.** (**A**) TMRM labelled mitochondria in Patients 1–3 fibroblasts (*top*), transduced with wild-type *STAT2* (*middle*), and *DRP1^−^*, *STAT1^−^* and healthy control (HC) cells (*bottom*); all cells at 70% confluency. Minus sign represents autosomal dominant mutation. (**B**) TMRM labelled mitochondria in SHSY5Y cells, wild-type (*left*) and *STAT2* knockout (*right*). (**C**) ImageJ quantitation of mitochondrial perimeter length in fibroblasts (*top*) and SHSY5Y cells (*bottom*). **P < 0.005.

**Figure 3:**
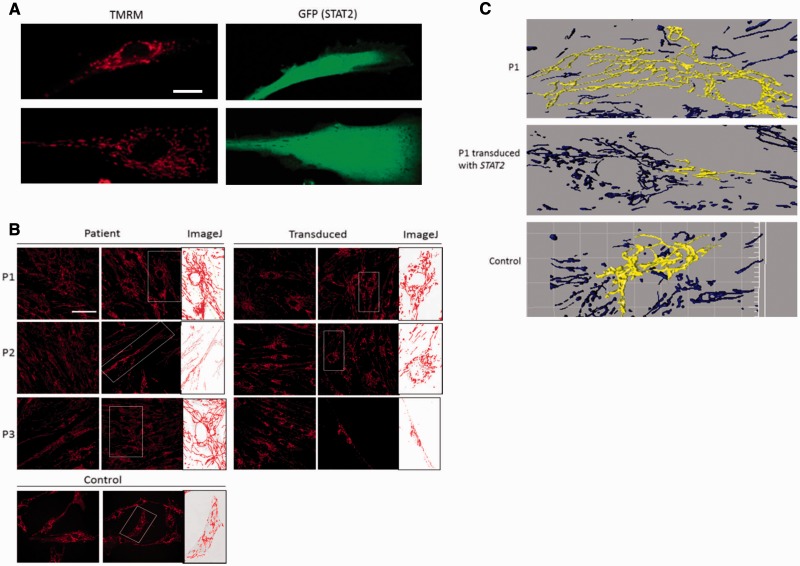
**Image analysis of mitochondria in transduced patient and control fibroblasts.** (**A**) Confirmation of lentiviral transduction in patient fibroblasts using GFP tag. Patient fibroblasts transduced with *STAT2*, stained with TMRM (left) and GFP expression upon reintroduction of *STAT2* (*right*). Scale bar = 20 μm. (**B**) TMRM stained mitochondria in *STAT2* transduced patient and control fibroblasts. Mito-Morphology Macro was installed on ImageJ, which measures mitochondrial interconnectivity and elongation from epifluorescence micrographs of cells stained for mitochondria. The perimeter length of the selected mitochondria is then measured using the software. Scale bar = 50 μm. (**C**) Three-dimensional representations of mitochondria in Patient 1 fibroblasts before (*top*) and after (*middle*) transduction with *STAT2*, and control fibroblasts (*bottom*). Confocal *z*-stack images of mitochondria stained with TMRM were reconstructed using IMARIS X64 software (version 7.6.3, Bitplane) to give 3D representations of the mitochondria present in each cell. Single connected mitochondria depicted as yellow.

Confocal microscopy demonstrated dense, elongated mitochondria in patient fibroblasts compared to controls in 80% confluent cell cultures (Fig. 2) We found that mitochondria from a clinically similar but unrelated patient (Patient 3), previously reported with a homozygous splice mutation in *STAT2* (c.381 + 5 G > C), were also elongated and tubular (Fig. 2), confirming the association between *STAT2* deficiency and mitochondrial elongation. Furthermore patient cells were transduced with wild-type *STAT2* and the delivery of the correct version of *STAT2* was confirmed by green fluorescent microscopy (Fig. 3A). Analysing the mitochondrial length using TMRM (Fig. 2) and quantitative methods such as ImageJ (Fig. 3B) and IMARIS X64 software (Fig. 3C) revealed an average 3-fold decrease in mitochondrial length in patient fibroblasts compared to controls following transduction with wild-type *STAT2.*

#### *STAT2* silencing

To confirm the specific role of STAT2 as a determinant of mitochondrial structure, SHSY5Y cells were transduced with siRNA targeting *STAT2.* Quantitative analysis showed a 4-fold increase in mitochondrial length in *STAT2*-knockout SHSY5Y cells compared to wild-type (Fig. 2B and C).

### Cellular analyses

#### Transcript expression

**Figure 4:**
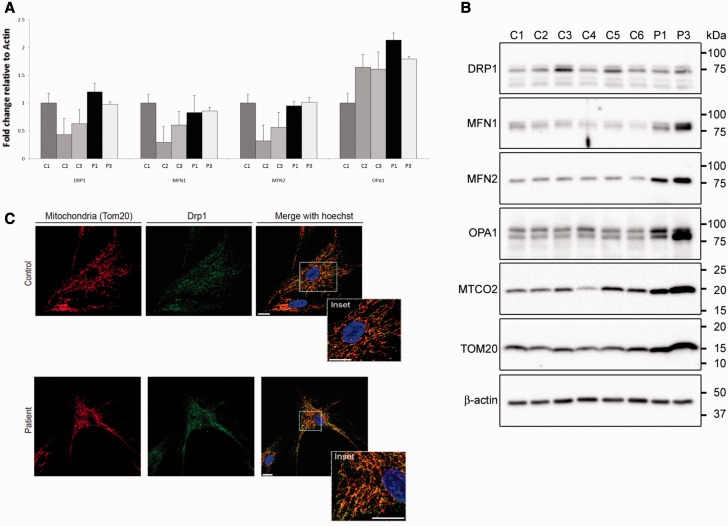
**Transcript and protein expression of fission and fusion proteins and DRP1 localization.** (**A**) Quantitative PCR analysis of DRP1, MFN1, MFN2 and OPA1 in three controls (C1–C3) and two patients (Patients 1 and 3). The samples were normalized to C1 and *ACTB* encoding β-actin was used as an endogenous control (*n = *3). (**B**) Western blot analyses of 14 μg protein extracted with 1.5% *n*-dodecyl-β-d-maltoside from control (C1–6) and patient (Patients 1 and 3) fibroblast cultures. Blots were probed with antibodies raised against DRP1, MFN1, MFN2, OPA1, MTCO2 and TOM20 as indicated. An antibody raised against β-actin served as loading control. (**C**) DRP1 localization was carried out by labelling the mitochondria with TOM20, DRP1 with FITC conjugated secondary antibody and nuclei with Hoechst and visualized under a confocal microscope. Overlaid images show DRP1 co-localization with TOM20 on the mitochondria.

We observed no difference in expression of genes encoding fission (*DNM1L*, also known as *DRP1*) and fusion (*MFN1*, *MFN2* and *OPA1*) proteins between patient and control fibroblasts (Fig. 4A).

#### Protein expression

**Figure 5:**
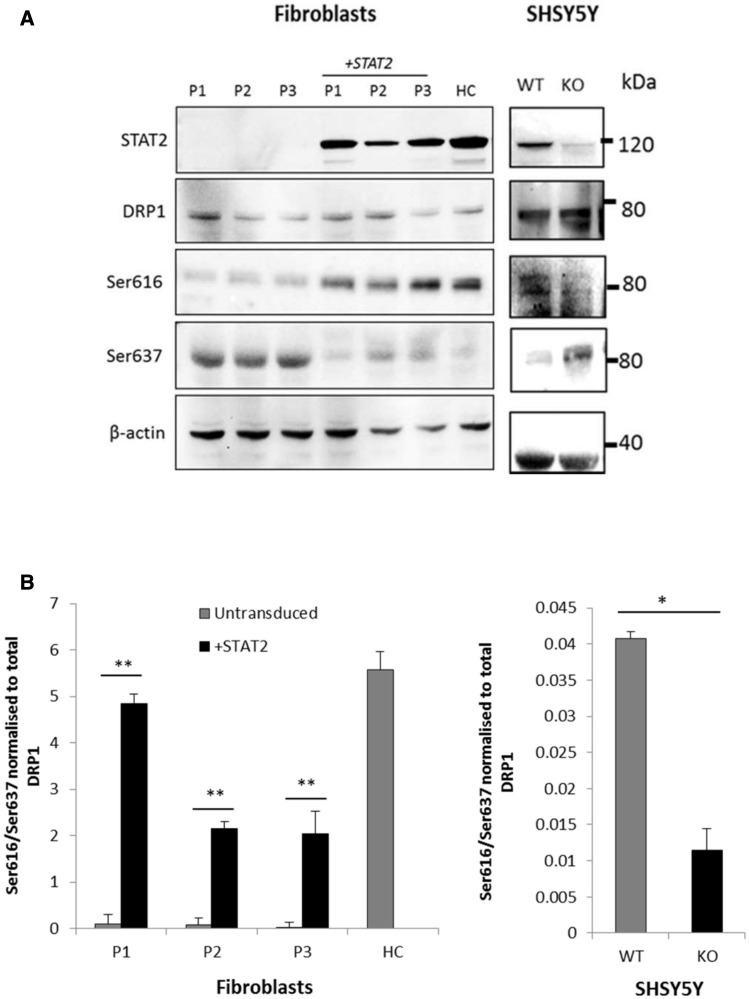
**DRP1 phosphorylation in patients and controls.** (**A**) Protein samples (20 μg) from fibroblasts and SHSY5Y cells were separated by sodium dodecyl sulphate polyacrylamide gel electrophoresis and immunoblotted with the indicated antibodies. STAT2 protein was not detectable in the three patients (P1–3) confirming nonsense mediated decay; STAT2 levels were restored after lentiviral transduction with *STAT2* (*top*). Total DRP1 protein levels remained constant throughout (*second panel from top*), but DRP1 phosphorylation at serine 616 was very low in STAT2 deficient cells and increased after lentiviral transduction with wild-type *STAT2* (*third panel*), whilst phosphorylation at serine 637 reduced after *STAT2* transduction (*fourth panel*). Actin was used as a loading control (*bottom*). (**B**) Quantitative analysis of phosphorylation at serines 616 and 637 of DRP1 in *STAT2* transduced patient and control fibroblasts (*left*) and SHSY5Y wild-type and *STAT2* knockout cells (*right*). Data were normalized to the total level of DRP1 and represent the mean ± SD of three independent experiments. *P < 0.05, **P < 0.005.

Western blot analysis revealed that STAT2 protein was undetectable in fibroblasts from all three patients Patients 1–3 (Fig. 5A), confirming nonsense mediated decay. We examined the effects of STAT2 deficiency on proteins involved in mitochondrial fission and fusion. Steady-state levels of DRP1 were similar in cytoplasmic protein extracts of cultured fibroblasts from Patient 1 and Patient 3, compared to six control subjects. However, the outer and inner mitochondrial membrane fusion proteins MFN1, MFN2 and OPA1 were increased in Patient 1 and Patient 3 compared to the six control fibroblasts (Fig. 4B). Further analyses demonstrated that the steady-state levels of mitochondrial cytochrome *c* oxidase subunit II (MT-CO2) and the translocase of outer mitochondrial membrane 20 (TOM20, encoded by *TOMM20*) were similarly increased in the patients (Fig. 4B).

#### DRP1 localization

For DRP1 to exert its effects on initiation of mitochondrial fission, it first needs to be localized to the mitochondria. Using confocal microscopy, we examined DRP1 localization in Patient 1 and control fibroblasts, and found DRP1 to be co-localized with the outer mitochondrial membrane protein TOM20 in both patient and control fibroblasts. Thus a deficiency of STAT2 protein does not appear to affect DRP1 localization to the mitochondria (Fig. 4C).

#### DRP1 phosphorylation

To further investigate the underlying cause of the long mitochondria, we measured activation of DRP1, the main fission factor in human mitochondria. As DRP1 levels and localization were normal in patient cells, we hypothesized that DRP1 might be inactive in patient cells and therefore unable to complete the fission process. DRP1 activity is dually regulated by phosphorylation at two key serine residues. Post-translational modification by phosphorylation at serine residue 616 (P-DRP1^S616^) is known to activate DRP1, whereas phosphorylation at serine 637 (P-DRP1^S637^) is associated with the inactive state of the GTPase. Reduced P-DRP1^S616^ and increased P-DRP1^S637^ was observed in all three patients compared to controls (Fig. 5), indicating that DRP1 is inactive in STAT2 deficiency. Introduction of wild-type *STAT2* into patient fibroblasts using lentiviral transduction reversed the phenotypes; we observed increased P-DRP1^S616^, decreased P-DRP1^S637^ and shorter mitochondria, reflecting a state of increased fission. Conversely, knock down of *STAT2* in SHSY5Y cells led to increased P-DRP1^S637^and reduced P-DRP1^S616^ (Fig. 5) and extremely elongated mitochondria (Fig. 2B), recapitulating the phenotype in STAT2-deficient fibroblasts.

#### STAT1 phosphorylation detected by fluorescence-activated cell sorting

**Figure 6:**
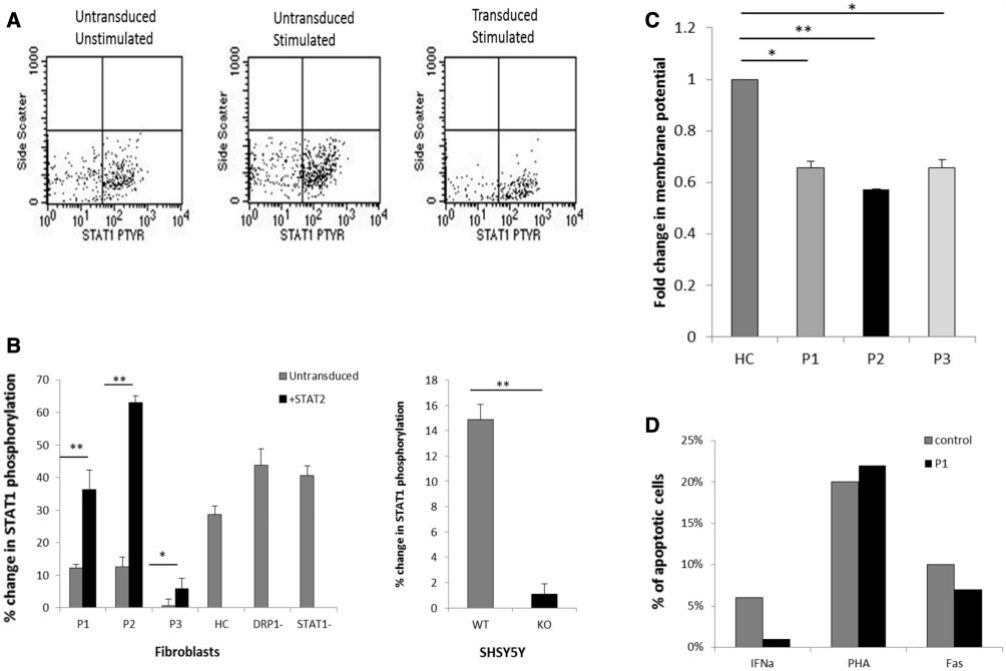
**STAT1 phosphorylation before and after stimulation with IFNα, membrane potential and apoptosis levels.** (**A**) Representative FACS dot blot graph showing phosphorylated STAT1 in *STAT2* transduced cells compared to non-transduced STAT2 deficient cells after stimulation (results for Patient 3 shown). (**B**) The percentage change in phosphorylated cells was calculated from the data from **A** [patient cells, transduced cells and SHSY5 wild-type (WT) and STAT2 knockout (KO) cells]. Data represents the mean ± SD (*n = *3). *P < 0.05, **P < 0.005 (**C**) Membrane potential analysis in control and patient cells using TMRM staining. The mean fluorescence intensity was analysed via confocal microscopy and Image J (*n = *3;>27 cells analysed for each *n*, ± SEM) *P < 0.05, **P < 0.005. (**D**) Apoptosis was analysed in fibroblasts derived from the STAT2 deficient patient (Patient 1) and compared to fibroblasts derived from healthy individuals. Cultured fibroblasts were left unstimulated or stimulated with 100 U/ml IFNα, 10 µg/ml anti-fasL IgM or phytohaemagglutinin. Cells were analysed using CellQuest Pro and the percentage of apoptotic cells defined as annexin V+7AAD+.

As well as STAT2, phosphorylation of another STAT protein called STAT1, was also disrupted in Patient 1 and Patient 2 fibroblasts compared to controls and recovered significantly after transducing the cells with wild-type *STAT2* (Fig. 6A and B), confirming disturbance of the IFNα pathway and explaining the susceptibility to viral infection in these patients.

#### Apoptosis assay

ImageJ analysis of TMRM stained fibroblasts revealed reduced mitochondrial membrane potential in all three patient fibroblasts Patients 1–3 (Fig. 6C). We therefore investigated apoptosis in Patient 1 cells compared to healthy controls. Induction with phytohaemagglutinin and anti-fasL both induced comparable apoptosis in patient and control cells. However, the STAT2 deficient cells failed to undergo apoptosis in response to IFNα (Fig. 6D). Both phytohaemagglutinin and anti-fasL induction of apoptosis resulted in the activation of caspase 3. These results indicate that there is not an inherent apoptotic defect in these cells but that IFNα induced apoptosis is severely impaired, consistent with previous observations in *STAT2* knockdown cell models (Romero-Weaver *et al.*, 2010).

## Discussion

We have found that STAT2, a component of the JAK-STAT cytokine signalling pathway, is a novel regulator of mitochondrial fission. This provides new insight into how the cell may orchestrate mitochondrial morphology and dynamics in concert with other cellular compartments. Modulation of mitochondrial shape can be detrimental to organelle function and can lead to a distinct shift in cell viability with unregulated fission reported in neurodegenerative disorders such as Alzheimer’s, Huntington’s and Parkinson’s diseases (Dagda *et al.*, 2009; Wang *et al.*, 2009; Kim *et al.*, 2010).

DRP1 is the master regulator of mitochondrial fission, though how it is ultimately recruited to the mitochondrial outer membrane remains largely unknown (Palmer *et al.*, 2011; Losón *et al.*, 2013). Complicating the issue further is the fact that DRP1 can undergo numerous post-translational modifications by the action of various proteins at different stages of the cell cycle. In this report, studies of mitochondrial morphology in three patients from two unrelated pedigrees with *STAT2* mutations revealed an elongated mitochondrial network. It appears that these patients harbour decreased levels of DRP1 phosphorylated at serine 616 (P-DRP1^S616^) and increased levels of DRP1 phosphorylated at serine 637 (P-DRP1^S637^). Both post-translational modifications render DRP1 largely inactive in the cytosol, unable to polymerise into higher order structures and assemble into the fission apparatus on the mitochondrial outer membrane—thus causing a block in the fission process (Taguchi *et al.*, 2007; Chang and Blackstone, 2010; Gomes *et al.*, 2011). Deducing what is a block in fission and what is stress induced hyperfusion is at times difficult; however, hyperfusion is most commonly observed as an acute response to stress and is a transient state before mitochondrial fragmentation (Gomes *et al.*, 2011).

We observed normal steady state levels of DRP1 and increased protein levels of the fusion proteins MFN1, MFN2 and OPA1 and also other mitochondrial proteins (MTCO2 and TOM20) compared to controls. This suggests that the relative mitochondrial hyperfusion, as a result of defective fission following DRP1 inactivation in STAT2 deficiency, leads to an increase in mitochondrial mass and results in raised levels of mitochondrial proteins, including MFN1, MFN2 and OPA1, but does not affect the levels of cytosolic proteins, such as DRP1. The increase in mitochondrial mass is further corroborated by the doubling in mtDNA copy number in cultured fibroblasts from Patient 1 (data not shown). Given the reduction in endogenous levels of P-DRP1^S616^ in these patients with *STAT2* mutations, it would seem that a modulation in the activity of DRP1 is directly responsible for the mitochondrial phenotype and results in defective fission.

Intercurrent infection frequently triggers severe decompensation in patients with mitochondrial disease. It has been assumed that the underlying energy defect limits the ability of the patient to cope with the added metabolic stress of infection. However, more recently, we have observed in our clinical practice that very specific infections can trigger fatal outcomes in patients who were not previously known to have mitochondrial disease prior to the viral illness. For example, we demonstrated that a previously well infant with overwhelming cardiomyopathy following respiratory syncytial virus infection had mutations in the *NDUFAF1* gene encoding an assembly factor of complex I, a key enzyme of OXPHOS (Fassone *et al.*, 2011). We also found mutations in another complex I assembly factor in another previously well infant who died of severe cardiomyopathy following parainfluenza infection (unpublished data). *STAT2*-knockout mice have severe susceptibility to viral infection, and STAT2 is a binding target for measles, a possible culprit in these patients (Park *et al.*, 2000; Rarnachandran *et al.*, 2008; Alazawi *et al.*, 2013).

In view of the endosymbiotic hypothesis (which proposes that mitochondria originate from bacteria which were engulfed by and subsequently developed an endosymbiotic relationship with the host eukaryotic cell (Margulis, 1975), it is not surprising that components of the innate immune system may also interact with, and perhaps even regulate, mitochondria. The data from this study suggest an unexpected link between innate immunity and mitochondrial function and suggest a potential new avenue for developing pharmacological strategies to treat mitochondrial diseases, for which virtually no effective curative treatments exist at present (Kanabus *et al.*, 2014).

Seven known mammalian STATs have been reported to be transcription factors with diverse biological functions, including immune regulation and development (Meier and Larner, 2014). Recently, non-canonical STAT functions have emerged, suggesting they play fundamental roles in cellular homeostasis (Meier and Larner, 2014). STAT3, the first to be found in mitochondria, has been implicated in the function of OXPHOS complexes I and II, suggesting that STAT3 is required for optimal energy production (Wegrzyn *et al.*, 2009). Basal pools of STAT1 and STAT2 have recently also been identified within mitochondria, and have been postulated to down regulate mitochondrial transcription (Goswami *et al.*, 2013; Lartigue and Faustin, 2013), possibly to co-ordinate the immune response and energy production. This is particularly interesting in light of a recent report that DRP1 may control T-cell activation at the immune synapse (Baixauli *et al.*, 2011). Previously cyclin B1-cyclin-dependent kinase and cAMP-dependent protein kinase A have been demonstrated to phosphorylate DRP1 on Ser616 and Ser637 respectively (Archer, 2013). We now suggest that STAT2 is an additional phosphorylase capable of phosphorylating DRP1 on Ser616.

**Figure 7:**
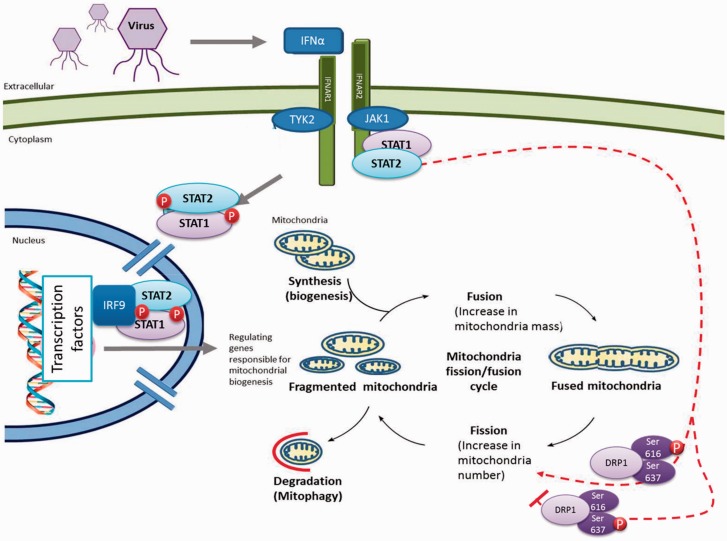
**Activation of the JAK-STAT pathway by viruses and the biogenesis, fission and fusion life cycle of mitochondria.** Viral infections activate a cascade of signals to produce cytokines, which then activate JAK-STAT proteins which then form a complex and translocate into the nucleus. The STAT1/2 dimer protein complex binds to IRF9 in the nucleus and the resulting trimer interacts with DNA at transcription factor binding sites, and upregulates gene expression. The dotted red line indicates effects of STAT2 on mitochondrial fission, mediated by DRP1 phosphorylation in healthy cells. Mitochondrial morphology is maintained by a balance of fission and fusion events, with disparity leading to a distinct shift in viability of the organelle, which can result in disease states. Phosphorylation of DRP1 at differing serine residues (Ser616 and Ser637) within the protein results in varied effects on DRP1 activity. Phosphorylation at Ser637 inhibits the GTPase activity of DRP1, leading to a defect in mitochondrial fission—promoting mitochondrial elongation.

In summary, STATs have increasingly recognized roles in mitochondrial function, and we have demonstrated that STAT2 is a novel regulator of mitochondrial fission (Fig. 7). Manipulation of the JAK-STAT pathway may represent a novel therapeutic strategy for mitochondrial diseases, by fine-tuning the balance of mitochondrial fission and fusion.

## Supplementary Material

Supplementary Data

Supplementary Data

## Abbreviations

DRP1: dynamin related protein 1
IFNα: interferon alpha
JAK: janus kinase
P-DRP1^S616^: phosphorylation at serine residue 616 of DRP1 (active DRP1)
P-DRP1^S637^: phosphorylation at serine 637 (inactive DRP1)
STAT: signal transduction and activation of transcription
TMRM: tetramethyl rhodamine methyl ester
