# Supplementary material for: Signal transducer and activator of transcription 2 deficiency is a novel disorder of mitochondrial fission
Source: Brain. 2015 Jun 30;138(10):2834–46. doi: 10.1093/brain/awv182 (PMC5808733; doi:10.1093/brain/awv182)
Supplement: Supplementary Data [file awv182_supplementary_data.zip › brain-2015-00180-File012.pdf]

Supplementary Table 1 Clinical data

**Parameter**	**Patient 1**	**Patient 2**	**Reference range**
Full blood count			
Haemoglobin	96	69	105–135 g/l
White blood cells	4.6	11.5	5.0–15.0 × 10^9/l
Neutrophils	2.9	11.3	1.5–8.5 × 10^9/l
Lymphocytes	1.2	0.1	2.0–9.5 × 10^9/l
Platelets	34	176	150–450 × 10^9/l
**Lymphocyte subsets**		11.5	
Total white cell count	5.21	5.07	5.0–15.0 × 10^9/l
Total lymphocytes	1.89	1.90	2.0–9.5 × 10^9/l
CD3	71.0%, 1.34 × 10^9/l	34.0%, 0.65× 10^9/l	43–76%
CD19	11.0%, 0.21 × 10^9/l	45.0%, 0.86× 10^9/l	14–44%
CD16+CD56+	14.0%, 0.26 × 10^9/l	12.0%, 0.23× 10^9/l	4–23%
CD3+CD56+	2.0%, 0.04 × 10^9/l	0.9%, 0.02 × 10^9/l	5–26%
CD3+CD4+	20.0%, 0.38 × 10^9/l	21.0%, 0.40 × 10^9/l	23–48%
CD3+CD8+	49.0%, 0.93 × 10^9/l	12.0%, 0.23 × 10^9/l	14–33%
**Spectratyping**			
CD4 Positive T Cells	0.2 × 10^6 cells/ml	0.4 × 10^6 cells/ml	–
CD8 Positive T Cells	0.4 × 10^6 cells/ml	0.2 × 10^6 cells/ml	
**TRECS**			
CD4 Positive T Cells	3008	397	>10000
CD8 Positive T Cells	0	130	TRECS/10^6 T Cells
**T memory panel**			
Naive: CD4+CD45RA+CD27+	42.0%	65.0%	50–85%
Memory: CD4+CD45RA-CD27+	51.0%	32.0%	53–86%
Effector:CD4+CD45RA+CD27-	0.3%	1.0%	0.42–1.5%
Naive:CD8+CD45RA+CD27+	13.0%	25.0%	69–97%
Memory:CD8+CD45RA-CD27+	23.0%	72.0%	53–86%
Effector:CD8+CD45RA+CD27-	47.0%	0.9%	0.26–0.85%
**Immunoglobulins**			
IgG	14.8	12.4	3.7–15.8 g/l
IgA	**0.12**	**0.13**	0.3–1.3 g/l
IgM	1.41	**0.39**	0.5–2.2 g/l
CRP	365	-	<20 mg/l

ESR	60	-	0.0–10.0 mm/h
**Liver function**			
Alanine aminotransferase	**239**	13	5–45 U/l
Gamma glutamyl transferase	**85**	-	6–19 U/l
Ammonia	**179**	-	<40 µmol/l
Prothrombin time	**12.5**	**12.9**	9.6–11.8 s
**Viral serology:**			
Measles IgM	1.89	0.115	0– 1.2 IU/ml
Measles IgG	1.55	2	0– 1.1 IU/ml
Mumps IgM	2.86	17.56	≤0.8 IU/ml
Mumps IgG	30.95	8.67	≤0.9 IU/ml
Rubella IgM	-	-	–
Rubella IgG	1.74	4.68	>15 IU/ml
**Autoantibodies:**			
Smooth muscle	negative	weak positive	–
Gastric parietal cell	negative	negative	–
Mitochondrial	negative	negative	–
Reticulin	negative	negative	–
Liver kidney microsomal	negative	negative	–
Anti-nuclear	negative	-	–
ANCA	0.5	-	0–1.99 IU/ml
Rheumatoid factor	**27**	-	0–20 IU/ml
Glomerular basement membrane	2	-	0–6.9 U/ml
Plasma lactate	**2.4**	1.7	<2.0 mmol/l
CSF lactate	2.6	1.1	<2.0 mmol/l
Plasma glucose	5.3	4.0	3.5–5.5 mmol/l
CSF glucose	1.7	2.2	–
CSF protein	0.39	<0.10l	0.15– 0.45 g/l
CSF WBC cell count	12	2	$<1 \times 10^6$/l
CSF oligoclonal bands	-	positive	–
CSF pterins			
Total Neopterin	**1095**	**173**	7–65 nmol/l
Tetrahydrobiopterin	36	26	8–57 nmol/l
Dihydrobiopterin	**42.8**	5.8	0.4–13.9 nmol/l
Plasma amino acids	*	Proline **376**	85–290 µmol/l
Urine organic acids	**Moderately raised pyruvate e × cretion**	-	-
Renal tubular function			
Albumin/creatinine	**18.2**	-	0.5–3.3 mg/mmol

NAG/creatinine	**416**	**176**	2–22 units/mmol
RBP/creatinine	83	11	4.5–89 µg/mmol
**Muscle respiratory chain enzyme complex activities**			
Complex I/CS	0.236	0.261	0.104–0.268
Complexes II+CIII/CS	0.196	0.076	0.040–0.204
Complex IV/CS	0.031	0.014	0.014–0.034

The following immunological and metabolic investigations were normal: perforin expression, cytotoxic granule release, complement C3 and C4, classical and alternative complement pathways, urinary catecholamines, biotinidase, transferrin electrophoresis, very long chain fatty acids, white cell enzymes, purines and pyrimidines, copper and caeruloplasmin

*Several amino acids low because of poor nutritional state; - = not available; NAG = *N*-acetylglucosaminidase; RBP = retinol binding protein; TRECS = T cell receptor excision circles; WBC = white blood cell
