# Supplementary figures and images for: Signal transducer and activator of transcription 2 deficiency is a novel disorder of mitochondrial fission
Source: Brain. 2015 Jun 30;138(10):2834–46. doi: 10.1093/brain/awv182 (PMC5808733; doi:10.1093/brain/awv182)

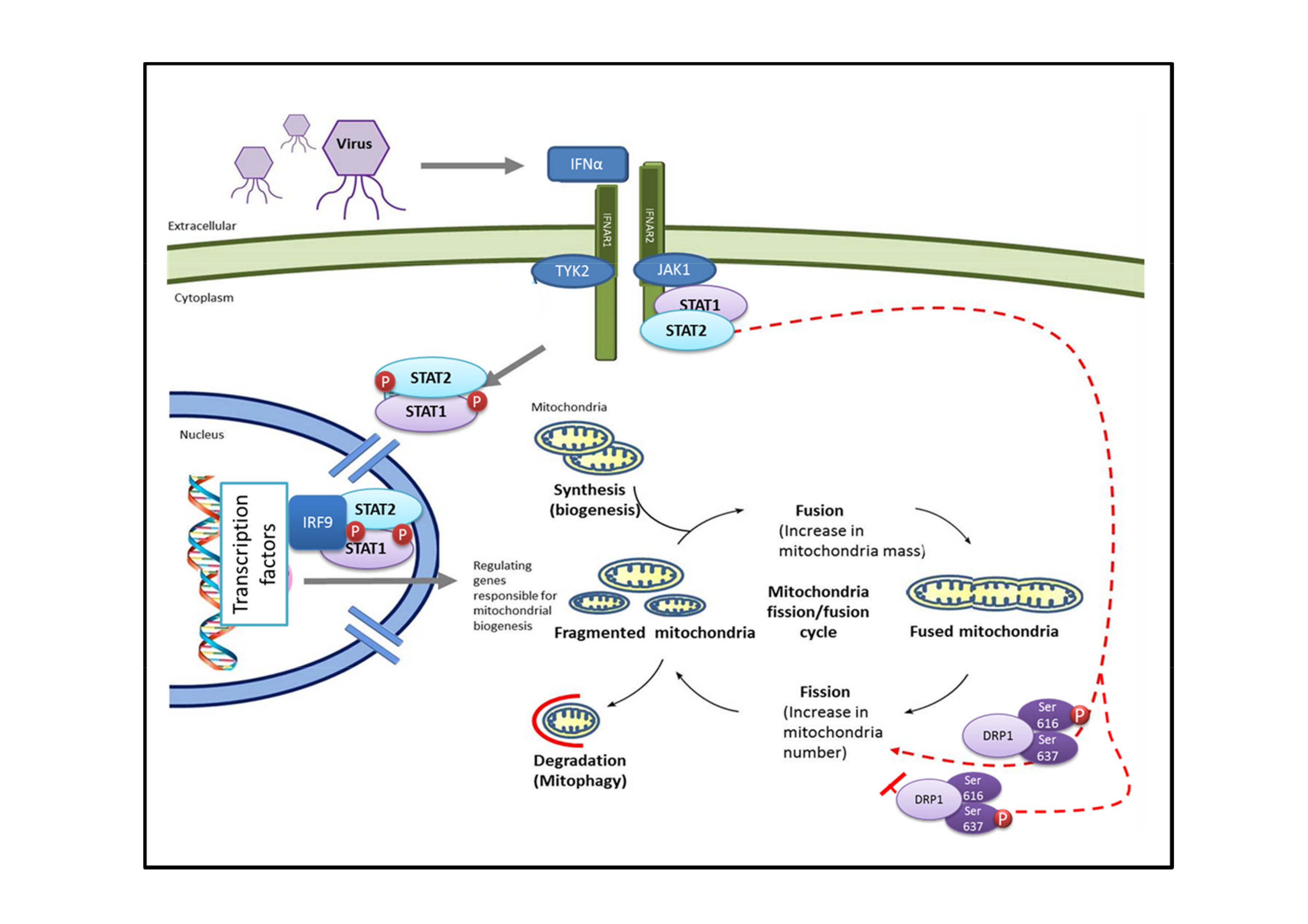

Supplement: Supplementary Data [file awv182_supplementary_data.zip › brain-2015-00180-File010.jpg]
